# Colchicum in Cholera

**Published:** 1831

**Authors:** 


### XXXIII.

### Colchicum in Cholera. By Mr. Taitt, Caen, Normandy.

The subject of cholera, from a dread of its appearance in this part of Europe, seems to occupy much public attention; it therefore becomes the duty of every professional man, to give his experience in the disease, so formidable in its nature, and so intractable in its management. With this view a communication was made to the Editors of the Edinburgh Medical Journal, in December last, by a young friend of mine, stating the successful use I have made of the saturated Tincture of Colchicum in many cases of the milder form of the cholera, as it appears in this part of Europe; but as the paper has not been acknowledged, I beg, if you think it of sufficient importance, that you will, through the medium of your Journal, suggest it as a remedy worthy of trial in the more aggravated forms of the disease, as it appears in Asia, and lately in Eastern Europe. It was administered in doses of from sixty to eighty drops; and in no case did it fail to produce immediate, decided, and lasting benefit; except in that of an infant, which was dying when I visited it first. I think it a valuable remedy in cases of indigestion, with vomiting and purging, as well as in diarrhœa proceeding from a deficiency or total want of bile; all which appear to be modifications of the same disease. There is at present residing in your neighbourhood a most respectable family who can vouch for its efficacy—six members of which, in common with many other inhabitants, both English and French, residing on the same side of a street in this city, were seized on the same night, some time in September, 1829, five of whom were relieved by this remedy, and up and well in a few hours, after a dose of castor oil—while two others, mother and daughter, who would not consent to take it, were confined several days. I will not enter into any theoretical speculations on the subject, but simply state, that it was in fine taken by myself, as a probable remedy, and powerful stimulus in exciting the liver, when in a state of great torpor, and aggravated by exposure to a cold evening air, in the vale of Taunton, after a fatiguing hot ride through part of Devonshire, during a sultry day in the month of Sept. 1825. My attack was ushered in by cold chills and sensations similar to those of a slight paroxysm of intermittent, followed, on awaking four or five hours afterwards, by frequent eructation of offensive gas, vomiting and purging. Being in the habit of taking this tincture to regulate the biliary secretion, which was deranged by a long residence in the West Indies, I happened to have a vial in my chaise-box, and took about sixty drops, by the light of the street lamp. This dose arrested the symptoms in less than fifteen minutes, and enabled me, after a dose of Seidlitz in the morning, to proceed on my journey. A similar effect followed an attack the next Autumn at Liverpool, when much of the disease at that time prevailed. I communicated these circumstances to a friend of mine, at present on the civil establishment at Madras, who not being a professional man, will no doubt have allowed them to escape him. He was, however, the father of the family above alluded to.

Your experience in both climates will enable you to judge of the probable success of this medicine in the diseases of India, if it be identified with that of Europe, or only differing in degree from accidental or particular causes; and your zeal I am sure will insure it a trial, if you think so.

R. Taitt.

*Caen, May*, 1831.

---

